# Supplementary material for: ProLanGO: Protein Function Prediction Using Neural Machine Translation Based on a Recurrent Neural Network
Source: Molecules. 2017 Oct 17;22(10):1732. doi: 10.3390/molecules22101732 (PMC6151571; doi:10.3390/molecules22101732)
Supplement: Supplementary file 1 [file molecules-22-01732-s001.pdf]

# Supplementary material

## - ProLanGO: protein function prediction using neural machine translation based on recurrent neural Network

Renzhi Cao, Colton Freitas, Leong Chan, Miao Sun, Haiqing Jiang, Zhangxin Chen

**Table S1** - The loss on training, testing dataset and training steps for k in range of 3 and 5.

| Loss on training dataset | Loss on testing dataset | Training steps |
|---|---|---|
| 1.79503328741 | 18.6675595748 | 4000 |
| 1.64131614149 | 18.4074899205 | 4400 |
| 1.597092987 | 16.5886364376 | 5200 |
| 1.34426462799 | 13.8441928341 | 5800 |
| 0.946846497655 | 13.7917128624 | 9400 |
| 0.910358985513 | 12.1674891888 | 9600 |
| 0.892778044641 | 11.7135422529 | 10600 |
| 0.832574125826 | 11.5991903582 | 11000 |
| 0.80839416191 | 9.56493561282 | 11800 |
| 0.380406224802 | 8.68890381275 | 32600 |
| 0.12263447782 | 8.62429404283 | 78400 |

**Table S2** - The loss on training, testing dataset and training steps for k in range of 3 and 6.

| Loss on training dataset | Loss on testing dataset | Training steps |
|---|---|---|
| 1.58393921524 | 18.4438511246 | 4400 |
| 1.59316055119 | 17.3700981774 | 4800 |
| 1.41222863525 | 15.0826662644 | 5000 |
| 1.24625749558 | 14.0610827033 | 6000 |
| 1.15679152817 | 13.2778996125 | 6600 |
| 1.07980198219 | 13.0642938216 | 7400 |
| 0.927364110053 | 12.475757805 | 9200 |
| 0.901390408576 | 11.6203115618 | 10000 |

| | | |
|---|---|---|
| 0.830895279795 | 11.5625016954 | 10800 |
| 0.751293131709 | 11.0157022742 | 12200 |
| 0.704609899372 | 9.45236195761 | 13200 |
| 0.373206133768 | 9.40182955377 | 34600 |
| 0.375746482685 | 9.35997710553 | 35400 |
| 0.315986161307 | 9.25985283725 | 46200 |

**Table S3** - The loss on training, testing dataset and training steps for k in range of 3 and 7.

| Loss on training dataset | Loss on testing dataset | Training steps |
|---|---|---|
| 1.73635725677 | 18.7370473775 | 60600 |
| 1.51168879539 | 17.7376708833 | 62200 |
| 1.5131638515 | 16.9422874468 | 62400 |
| 1.42100558966 | 15.1843712517 | 63400 |
| 1.30290189803 | 15.0542001756 | 64600 |
| 1.25596610814 | 14.1795183796 | 65600 |
| 1.20681206822 | 14.0169454495 | 66600 |
| 1.14389783055 | 12.7391165767 | 67800 |
| 1.10072250754 | 12.3808471847 | 68800 |
| 1.09540014923 | 10.9575151981 | 69000 |
| 0.855134223849 | 10.5491815639 | 75600 |
| 0.828636280149 | 9.80934950507 | 77600 |
| 0.755376954973 | 9.72645122932 | 81200 |
| 0.618407645822 | 9.45746709609 | 87400 |
| 0.577865229398 | 9.03719444237 | 92800 |
| 0.551491698697 | 8.5924420968 | 93800 |

**Table S4** - Reference papers of protein function prediction methods that are not used in this manuscript.

| Website link | Reference |
|---|---|
| NA | Hierarchical classification of gene ontology terms using the gostruct method (Sokolov and Ben-Hur 2010) |
| Software on Mac only | Parametric Bayesian priors and better choice of negative examples improve protein function prediction (Youngs et al. 2013) |
| http://sifter.berkeley.edu/ | SIFTER search: a web server for accurate phylogeny-based protein function prediction(Sahraeian, Luo, and Brenner 2015) |

| | |
|---|---|
| http://microserf.biocomp.unibo.it/bar/ | The bologna annotation resource: a non hierarchical method for the functional and structural annotation of protein sequences relying on a comparative large-scale genome analysis(Bartoli et al. 2009) |
| http://gorbi.irb.hr/ | Phyletic profiling with cliques of orthologs is enhanced by signatures of paralogy relationships(Skunca et al. 2013) |
| http://d2p2.pro/search | D(2)P(2): database of disordered protein predictions(Oates et al. 2012) |
| http://supfam.org/SUPERFAMILY/cgi-bin/dcpredictormain.cgi | A domain-centric solution to functional genomics via dcGO predictor.(Fang and Gough 2013) |
| http://supfam.org/SUPERFAMILY/hmm.html | Superfamily 1.75 including a domain-centric gene ontology method(de Lima Morais et al. 2011) |
| http://dragon.bio.purdue.edu/ESG | ESG: extended similarity group method for automated protein function prediction(Chitale et al. 2009) |
| http://www.cathdb.info/search/by_sequence | Functional classification of CATH superfamilies: a domain-based approach for protein function annotation(Das et al. 2016) |
| http://gofdr.tianlab.cn/ | GoFDR: a sequence alignment based method for predicting protein functions(Gong, Ning, and Tian 2016) |
| http://www.medcomp.medicina.unipd.it/Argot2/ | Argot2: a large scale function prediction tool relying on semantic similarity of weighted gene ontology terms(Falda et al. 2012) |
| http://protein.bio.unipd.it/inga | INGA: protein function prediction combining interaction networks, domain assignments and sequence similarity(Piovesan et al. 2015) |
| http://firedb.bioinfo.cnio.es | FireDB: a compendium of biological and pharmacologically relevant ligands(Maietta et al. 2014) |
| NA | A combined approach for genome wide protein function annotation/prediction(Benso et al. 2013) |
| NA | A fast ranking algorithm for predicting gene functions in biomolecular networks.(Re M 2017) |
| NA | Bayesian Markov Random Field Analysis for Protein Function Prediction Based on Network Data(Kourmpetis et al. 2010) |
| http://www.sbg.bio.ic.ac.uk/~mwass/combfunc/help.html | CombFunc: predicting protein function using heterogeneous data sources(Wass, Barton, and Sternberg 2012) |

## References:


Bartoli, Lisa, Ludovica Montanucci, Raffaele Fronza, Pier Luigi Martelli, Piero Fariselli, Luciana Carota, Giacinto Donvito, Giorgio P. Maggi, and Rita Casadio. 2009. "The Bologna Annotation Resource: A

Non Hierarchical Method for the Functional and Structural Annotation of Protein Sequences Relying on a Comparative Large-Scale Genome Analysis." *Journal of Proteome Research* 8 (9): 4362–71.

Benso, Alfredo, Stefano Di Carlo, Hafeez ur Rehman, Gianfranco Politano, Alessandro Savino, and Prashanth Suravajhala. 2013. "A Combined Approach for Genome Wide Protein Function Annotation/prediction." *Proteome Science* 11 (Suppl 1). BioMed Central: S1.

Chitale, Meghana, Troy Hawkins, Changsoon Park, and Daisuke Kihara. 2009. "ESG: Extended Similarity Group Method for Automated Protein Function Prediction." *Bioinformatics* 25 (14): 1739–45.

Das, Sayoni, David Lee, Ian Sillitoe, Natalie L. Dawson, Jonathan G. Lees, and Christine A. Orengo. 2016. "Functional Classification of CATH Superfamilies: A Domain-Based Approach for Protein Function Annotation." *Bioinformatics* 32 (18): 2889.

Falda, Marco, Stefano Toppo, Alessandro Pescarolo, Enrico Lavezzo, Barbara Di Camillo, Andrea Facchinetti, Elisa Cilia, Riccardo Velasco, and Paolo Fontana. 2012. "Argot2: A Large Scale Function Prediction Tool Relying on Semantic Similarity of Weighted Gene Ontology Terms." *BMC Bioinformatics* 13 Suppl 4 (March): S14.

Fang, Hai, and Julian Gough. 2013. "A Domain-Centric Solution to Functional Genomics via dcGO Predictor." *BMC Bioinformatics* 14 Suppl 3 (February): S9.

Gong, Qingtian, Wei Ning, and Weidong Tian. 2016. "GoFDR: A Sequence Alignment Based Method for Predicting Protein Functions." *Methods* 93 (January): 3–14.

Kourmpetis, Yiannis A. I., Aalt D. J. van Dijk, Marco C A, Roeland C. H. J. van Ham, and Cajo J. F. ter Braak. 2010. "Bayesian Markov Random Field Analysis for Protein Function Prediction Based on Network Data." *PloS One* 5 (2). Public Library of Science: e9293.

Lima Morais, David A. de, Hai Fang, Owen J. L. Rackham, Derek Wilson, Ralph Pethica, Cyrus Chothia, and Julian Gough. 2011. "SUPERFAMILY 1.75 Including a Domain-Centric Gene Ontology Method." *Nucleic Acids Research* 39 (Database issue): D427–34.

Maietta, Paolo, Gonzalo Lopez, Angel Carro, Benjamin J. Pingilley, Leticia G. Leon, Alfonso Valencia, and Michael L. Tress. 2014. "FireDB: A Compendium of Biological and Pharmacologically Relevant Ligands." *Nucleic Acids Research* 42 (Database issue). Oxford University Press: D267.

Oates, M. E., P. Romero, T. Ishida, M. Ghalwash, M. J. Mizianty, B. Xue, Z. Dosztanyi, et al. 2012. "D2P2: Database of Disordered Protein Predictions." *Nucleic Acids Research* 41 (D1): D508–16.

Piovesan, Damiano, Manuel Giollo, Emanuela Leonardi, Carlo Ferrari, and Silvio C. E. Tosatto. 2015. "INGA: Protein Function Prediction Combining Interaction Networks, Domain Assignments and Sequence Similarity." *Nucleic Acids Research* 43 (W1): W134–40.

Re M, Et al. 2017. "A Fast Ranking Algorithm for Predicting Gene Functions in Biomolecular Networks. - PubMed - NCBI." Accessed October 10. https://www.ncbi.nlm.nih.gov/pubmed/23221088.

Sahraeian, Sayed M., Kevin R. Luo, and Steven E. Brenner. 2015. "SIFTER Search: A Web Server for Accurate Phylogeny-Based Protein Function Prediction." *Nucleic Acids Research* 43 (W1): W141–47.

Skunca, Nives, Matko Bošnjak, Anita Kriško, Panče Panov, Sašo Džeroski, Tomislav Smuc, and Fran Supek. 2013. "Phyletic Profiling with Cliques of Orthologs Is Enhanced by Signatures of Paralogy Relationships." *PLoS Computational Biology* 9 (1): e1002852.

Sokolov, Artem, and Asa Ben-Hur. 2010. "Hierarchical Classification of Gene Ontology Terms Using the GOstruct Method." *Journal of Bioinformatics and Computational Biology* 8 (2): 357–76.

Wass, Mark N., Geraint Barton, and Michael J. E. Sternberg. 2012. "CombFunc: Predicting Protein Function Using Heterogeneous Data Sources." *Nucleic Acids Research* 40 (Web Server issue): W466–70.

Youngs, Noah, Duncan Penfold-Brown, Kevin Drew, Dennis Shasha, and Richard Bonneau. 2013. "Parametric Bayesian Priors and Better Choice of Negative Examples Improve Protein Function

Prediction." *Bioinformatics* 29 (9): 1190–98.
